# Nucleic acid triggers of autoimmunity and autoinflammation

**DOI:** 10.1016/j.coi.2025.102535

**Published:** 2025-01-30

**Authors:** Kaiyuan Hao, Ann Marshak-Rothstein

**Affiliations:** Department of Medicine, UMass Chan Medical School, Worcester, MA 01604, USA

## Abstract

The key role of nucleic acid sensing receptors in the development of autoimmune and autoinflammatory diseases is becoming increasingly apparent. Activation of these sensors has been attributed to the failure of professional scavenger cells to adequately clear cell debris, in many cases due to defective scavenger receptors. However, as now summarized in this review, numerous gain-of-function mutations in the nucleic acid sensing receptors, or in molecules that regulate sensor activity, have now been evaluated in gene-targeted murine strains, and critical components of their downstream pathways have been identified as therapeutic targets. In addition, we are beginning to understand how DNases and RNases play crucial roles in both generating and eliminating the distinct ligands that engage the various nucleic acid sensors. Murine models of disease have further provided important insights regarding the function of and synergy between individual endosomal and cytosolic receptors, as well as cell type restricted functions.

## Introduction

Mammalian endosomal Toll-like receptors (TLRs) were originally defined as sensors that could discriminate microbial nucleic acids (NAs) from mammalian NAs and thereby play a critical role in host defense [1]. However, since the initial identification of TLR9 as a sensor for bacterial DNA, the TLR family now includes endosomal RNA-sensing receptors TLR3, TLR7, TLR8, and TLR13. Cytosolic sensors for both DNA, such as absent in melanoma 2 (AIM2) and the cyclic guanosine monophosphate (cGMP)–AMP synthase (cGAS)–stimulator of interferon genes (STING) pathway, and RNA, such as melanoma differentiation–associated protein 5 (MDA5) and retinoic acid–inducible gene I have also been identified. We also know that these same sensors detect both mammalian and microbial NAs and that inappropriate activation of these receptors can contribute to a wide assortment of autoimmune and autoinflammatory diseases. Nevertheless, we are only beginning to appreciate the pivotal receptors, critical cell types, and downstream pathways responsible for the clinical manifestations that distinguish numerous NA-driven diseases from one another. Recent studies have focused on the specific roles of endosomal and cytosolic receptors in autoimmune and autoinflammatory diseases, as well as on the importance of nucleases in the degradation and generation of NA ligands.

### Importance of endosomal nucleic acid sensors in systemic lupus erythematosus and other autoimmune diseases

#### Toll-like receptor 7 in the development of systemic lupus erythematosus

One of the defining features of systemic lupus erythematosus (SLE) is the production of anti-DNA antibodies, dependent on B cell expression of TLR9 [2]. Nevertheless, murine models have continued to show a key role for TLR7 in the development of SLE, including the production of autoantibodies that bind RNA or RNA-associated proteins [2]. Evidence includes increased severity of SLE in mice that overexpress TLR7, dramatically improved status of TLR7-deficient SLE-prone mice, and a pivotal role for TLR7 in the development of a key subset of autoantibody producing B cells, often referred to as age-associated B cells or double-negative B cells [3–6]. The importance of TLR7 in lupus pathogenesis has been further validated by the more recent identification of numerous gain-of-function (GOF) variants of TLR7 and Unc93B1 (required for proper endosomal TLR trafficking) in SLE patients [7–10].

#### The Toll-like receptor 9 paradox in the development of systemic lupus erythematosus

**Figure 1:**
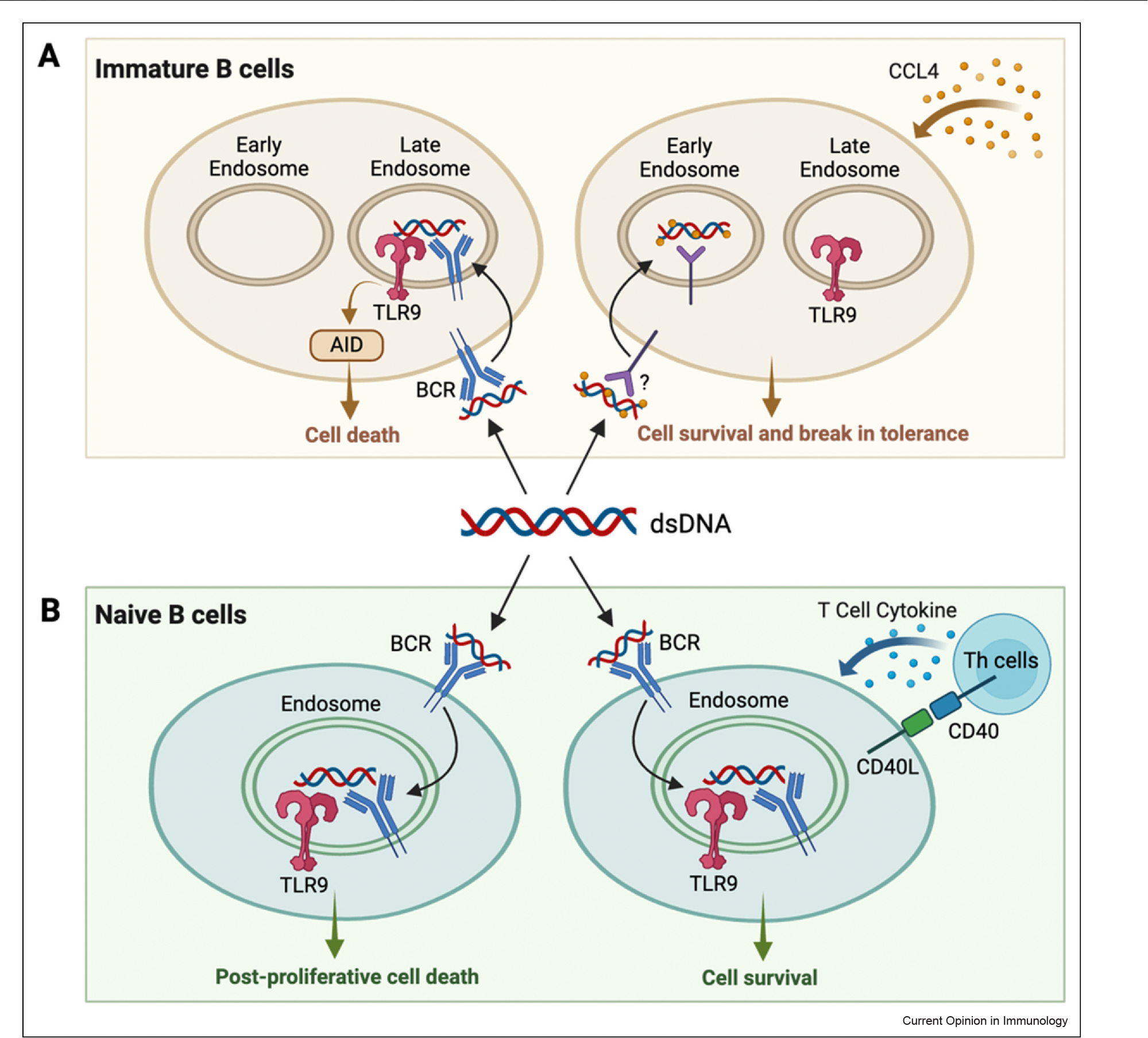
TLR9 signal is critical for establishing B cell tolerance. (a) In immature B cells, dsDNA internalized through BCR traffics to the late endosomes where TLR9 resides. TLR9 activation by internalized ligands leads to AID-mediated cell death. In the presence of chemokine CXCL4, CXCL4 complexes with dsDNA, and sequestered the ligands in the early endosomes, thus avoiding the TLR9 signal that is required for central tolerance. (b) In immature B cells, dsDNA internalized through BCR engages TLR9 and elicits a strong TLR9 signal that leads to proliferative cell death. However, upon CD40-CD40L interaction or receiving cytokines from T helper cells, the cell survives and potentially becomes autoreactive. Created in BioRender. Hao, K. (2025) https://BioRender.com/n68k519.

In contrast, TLR9-deficient mice often develop more severe, instead of less severe SLE [2,4], and over-expression of TLR9 in murine B cells ameliorates renal disease [11]. This paradox reflects a unique role for TLR9 in the elimination of potentially pathogenic autoreactive B cells during early B cell development in the bone marrow [12]. This process depends on trafficking of the TLR9 ligand to late endosomal compartments where TLR9 activation induces cell death through production of activation-induced cytidine deaminase (AID) [13]. CCL4 diverts ligand trafficking to an early TLR9^neg^ endosome and thereby prevents tolerance induction (Figure 1a) [12]. Alternatively, in mature naïve B cells, co-engagement of TLR9 and the B cell receptor (BCR), not BCR/TLR7 activation, induce a proliferative response that is followed by G1 arrest and subsequent apoptotic cell death [14,15]. However, these BCR/TLR9-stimulated cells can be rescued by CD40L or T cell cytokines (Figure 1b) [16] and, in the context of an ongoing TLR7-driven response, may then go on to produce pathogenic autoantibodies.

Moreover, in contrast to TLR7 and Unc93B1, GOF variants of TLR9 have not yet been identified in SLE patients. Murine studies have shown that forced overexpression of TLR9 in neonatal mice, but not adult mice, leads to fatal inflammation, likely due to the exceedingly high expression levels of TLR9 in specific subsets of neonatal inflammatory myeloid cells [17]. Thus, excessive TLR9 activity may be a developmental roadblock.

#### The SLC15A4/TASL/IRF5 complex in lupus pathogenesis

There are, nevertheless, parallels in TLR7 and TLR9 activation. Both pathways depend on interferon regulatory factor 5 (IRF5), and IRF5 activation depends on recruitment to an endolysosomal scaffold composed of the transport protein solute carrier family 15 member 4 (SLC15A4) and the TLR adaptor interacting with SLC15A4 (TASL) [18,19]. SLC15A4-deficiency completely rescued NZB/W SLE-prone mice from autoimmune disease [20], and highly effective small molecule inhibitors of the SLC15A/TASL complex have now been identified that could lead to the development of novel SLE therapeutics [21]. The potential importance of IRF5-directed therapeutics is further supported by recent studies in murine lupus where a 50% reduction in the level of IRF5, either before or after the onset of clinical disease, can dramatically reduce disease parameters [22,23].

#### The emerging role of Toll-like receptor 13 in the detection of single-stranded RNA

The less well-studied single-stranded ssRNA sensing murine receptor, TLR13, has also recently been linked to autoimmune disease. TLR13 binds a 13-nucleotide sequence within bacterial 23S ribosomal RNA [24]. Human TLR8 also specifically detects bacterial RNA as well as a related sequence in mitochondrial RNA [25]. Neutrophil extracellular traps (NETs) are a well-documented source of mammalian DNA but also contain substantial amounts of RNA [26], presumably including mitochondrial RNA, that when complexed with LL37, can induce dendritic cell (DC) production of proinflammatory cytokines. TLR13 is also highly expressed in neutrophils where it induces the further release of NETs and thereby promote chronic inflammation. Recent studies have indicated that LL37/RNA complexes, detected by either murine TLR13 or human TLR8, promote psoriasis [27,28] and may also contribute to other DC- or neutrophil-mediated inflammatory diseases.

### Gain-of-function mutations in cytosolic nucleic acid sensors promote autoinflammatory diseases

#### Stimulator of interferon genes gain-of-function mutations impact hematopoietic and nonhematopoietic tissues

As in the case of TLR7 and Unc93B1, patients with GOF mutations in cytosolic sensors can develop inflammatory disorders, and disease mechanisms have been explored in the homologus gene–targeted mice. For example, mice that express GOF mutation in STING recapitulate the interstitial lung disease (ILD) described in patients with STING-associated Vasculopathy in Onset in Infancy (SAVI) [29,30]. SAVI mice also develop a form of inflammatory bowel disease (IBD) [31]. Reconstitution of lethally irradiated SAVI mice with wild-type stem cells, and vice versa, revealed a critical role for hematopoietic cells in the initiation of IBD but not for ILD, where radioresistant nonhematopoietic cells were necessary and sufficient for lung inflammation. Conditional knock-in (CKI) SAVI mice have been used to direct expression of mutant allele to specific cell types where expression levels of the inserted gene remain under the control of the endogenous STING promoter. By crossing these SAVI CKI mice with tissue-specific Cre lines, it was found that expression of the mutant allele in myeloid cells was sufficient for the development of IBD, while expression in endothelial cells (ECs), but not hematopoietic, fibroblast, or epithelial cells, was sufficient for lung inflammation [32]. A separate study reported that EC-intrinsic hyperactivation of cGAS induced vascular ECs to produce CCL5, thereby recruiting T cells to the lung [33]. Furthermore, a recent study in multiple sclerosis patients found that STING preferentially accumulated in capillary ECs in the diseased areas of the brain, where it signaled through the TBK1-IRF3 axis to drive IFNb production that then perpetuated neuroinflammation [34]. These data demonstrate how cytosolic NA sensors in either hematopoietic or stromal cells can play critical roles in tissue-specific autoinflammatory conditions.

#### Gain-of-function mutations in melanoma differentiation–associated protein 5 promote numerous forms of autoinflammation

The cytosolic RNA sensor MDA5 is a susceptibility gene associated with a wide range of autoimmune diseases, now known to depend on elevated levels of type I interferon (IFN-I) [35]. Rare GOF mutations in MDA5 have been linked to a neuroinflammatory disease known as Aicardi-Goutieres syndrome (AGS) as well as a disorder associated with abnormal vascular and bone calcification known as Singleton-Merten syndrome. In mice, overexpression of MDA5 leads to increased production of IFN-I and exacerbates SLE pathology in lupus-prone strains [36]. Mice expressing an MDA5 GOF mutation also develop neuroinflammation and bone abnormalities [37,38] and are likely to provide a useful experimental tool for a better mechanistic understanding of these disorders.

### Regulation of nucleic acid sensor activation by DNA and RNA nucleases

#### Inability to degrade extracellular DNA can promote systemic lupus erythematosus and additional autoimmune diseases

Cell death and NETosis often result in release of endogenous NAs into the extracellular space, and their disposal relies on the activity of secreted nucleases. The DNase I family, including DNase I and DNase1L3, is the most extensively studied extracellular nucleases [39]. While both DNase I and DNase1L3 are capable of degrading naked DNA and NET-associated DNA [40], DNase1L3 is particularly adept at digesting DNA within intact chromatin and apoptotic microparticles [41]. Ineffective clearance of extracellular NAs due to malfunction of these nuclease can result in excessive immune cell efferocytosis and NA sensor engagement. Indeed, mice deficient in DNase1L3 develop anti-dsDNA and antinucleosome antibodies and an SLE-like disease that depends on both TLR7 and TLR9 [42]. It follows that individuals with genetic defects in DNase I and DNASE1L3 or circulating autoantibodies against DNase I and DNase1L3 are more prone to develop SLE, as well as arthritis, systemic sclerosis, and hidradenitis suppurativa [43–46], implicating DNA sensors in autoimmune conditions beyond lupus.

#### Excessive accumulation of intracellular nucleic acids can drive neuroinflammation

Intracellular NAs come from a variety of sources, including destabilized mitochondria, micronuclei, or retroelements (reviewed in Ref. [47]). While processes that generate excessive accumulation of NAs are frequently associated with infection or inflammation, normal cell turnover and cell physiology also generate DNA and RNA fragments that can contribute to severe autoinflammatory disorders. Successful removal of intracellular NA debris depends on intracellular nucleases whose loss can lead to severe autoinflammation.

AGS is an inherited encephalopathy in children, mentioned above, that is associated with calcifications of the basal ganglia and central nervous system (CNS) lymphocytosis [48]. The genetic basis of this disease was first revealed in 2006 as a loss-of-function (LOF) mutation in the cytosolic DNA exonuclease Trex1 [49], leading to the accumulation of undergraded DNA in the cytosol. A mouse model of Trex1 deficiency subsequently implicated the cytosolic cGAS/STING pathway in AGS pathogenesis [50]. Defects in numerous other genes involved RNA sensing (e.g. MDA5, as described above), and NA metabolism (e.g. RNaseH2, SAMHD1, and ADAR1) are now known to cause AGS and serve as key examples of diseases classified as type I interferonopathies [51]. Although mouse models of these mutations have pointed to aberrant activation of DNA or RNA sensors and the production of type I IFNs as a contributing factor in these disorders, homologous murine mutations often fail to develop CNS inflammation. Discrepancies may reflect the need for synergistic interactions between more than one dysregulated receptor or other immunoregulatory protein.

Cystic leukoencephalopathy (CLE) is another example of an autosomal recessive CNS disorder of children that results from an RNase deficiency, in this case, RNaseT2 [52]. RNaseT2^−/−^ mice recapitulate many of the neurological symptoms and also develop severe multiorgan inflammation. RNaseT2-deficient mice can be rescued by intercrossing with mice that are IFNαR deficient [53], again identifying CLE as a type I interferonopathy.

#### Endosomal DNase II deficiency impacts multiple tissues by activating both endosomal and cytosolic nucleic acid sensors

Patients with biallelic hypomorphic mutations in DNase II present with deforming arthropathy, splenomegaly, hepatic and renal inflammation, antinuclear autoantibodies (ANAs), and elevated serum levels of both type I and type II IFNs [54]. Since DNase II is an endolysosomal nuclease, TLR9 should be the NA sensor responsible for the resulting systemic inflammation. However, in mice, DNase II defciency is embryonic lethal due to the activation of the cytosolic DNA-sensing pathway cGAS/STING, not TLR9. DNase II^−/−^ embryos can be rescued by intercrossing with mice that lack a type I IFN receptor, but DNase II^−/−^ IFNaR^−/−^ double knockout mice (DKO) then develop an inflammatory arthritis, dependent on STING and AIM2 and not TLR9 [55,56]. DKO mice also develop an early-onset lupus-like phenotype, characterized by ANA production, splenomegaly, and lymphocyte activation. In addition, they develop liver pathology associated with portal inflammation and interstitial fibrosis. Intriguingly, these early features of systemic autoimmunity are not STING dependent. Rather, ANA production depends on TLR7, while splenomegaly, other lupus-like symptoms, and the liver inflammation depend on TLR9 [57,58].

Since DKO mice are IFNaR deficient, inflammation in this model is not type I IFN dependent. However, both the lupus-like symptoms and the liver inflammation are highly dependent on IFNg [57,58]. Overall, this model suggests that therapeutics directed at NA sensor–driven diseases may need to consider multiple NA sensors that differentially contribute to tissue-specific inflammatory responses.

#### Endosomal exonucleases phospholipase D3 and phospholipase D4 regulate ligand availability for Toll-like receptor 9 and RNA sensors

**Figure 2:**
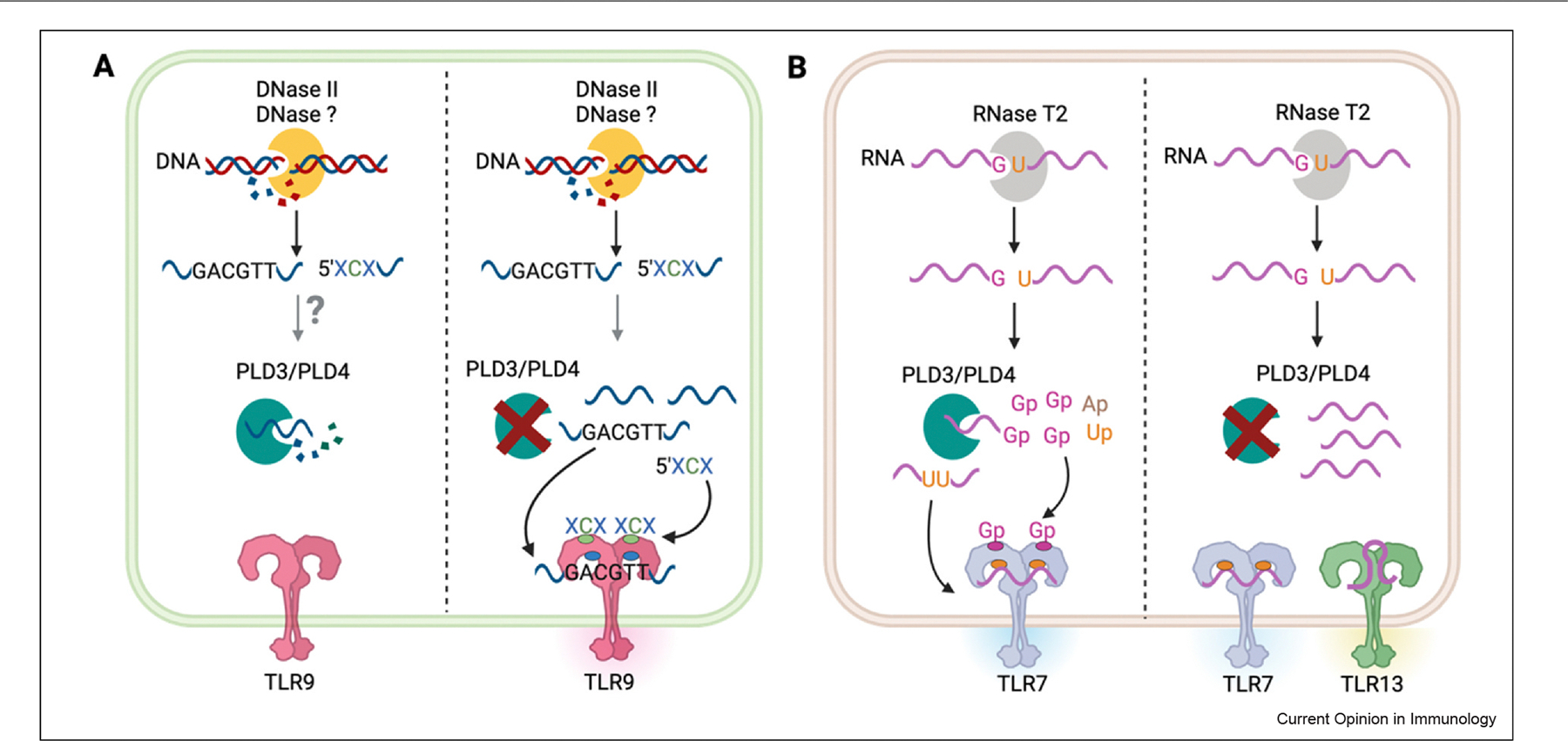
Processing of TLR7 and TLR9 ligands by endosomal nucleases. **(a)** In the endosome, long dsDNA is digested by DNase II or other undefined DNases into fragments with 5’-XCX motifs to fit binding site 1 (green) of TLR9, while DNA fragments with CpG motifs will engage binding site 2 (blue) of TLR9. Under homeostatic conditions, PLD3/ PLD4 function to degrade the DNA fragments into nucleotides, thus preventing aberrant TLR9 activation induced autoinflammation. **(b)** RNase T2 cleaves RNA between guanosine and uridine. RNaseT2-digested products are then processed by exonucleases PLD3/PLD4 to liberate the 2′,3′-cGMP that fit site 1 (pink) of TLR7. Left-over RNA fragments with uridine rich region engages with site 2 (orange) of TLR7. In cells deficient for PLD3^−/−^ PLD4^−/−^, excessive RNA and DNA fragment accumulates and potentially engages the TLR13 and the lower binding site of TLR7. Created in BioRender. Hao, K. (2025) https://BioRender.com/o32q277.

Members of the phospholipase D (PLD) family, PLD3 and PLD4, are endolysosomal exonucleases that can trim the 5’ end from both ssRNA and ssDNA fragments. Genome-wide studies connected PLD3 variants with susceptibility to neurodegenerative diseases and PLD4 variants to SLE and arthritis [59,60]. More precise molecular mechanisms of pathogenesis came from PLD-deficient mouse strains. PLD4 deficiency in B6 mice results in splenomegaly and immune abnormalities that resemble human macrophage activation syndrome and can be rescued by loss of either TLR9 or, to some extent, by IFNγ [61]. Although PLD3^−/−^ mice do not display an overt inflammatory phenotype, macrophages from PLD3^−/−^ mice respond more robustly to TLR9 ligands than controls, Importantly, mice deficient in both PLD3 and PLD4 develop even more severe macrophage activation syndrome-like autoinflammation than the PLD4 single KO mice and die from TLR-triggered hepatic damage in early life [62]. Short fragments of both DNA and RNA accumulate in the PLD3/PLD4 double-deficient mice and are proposed to cause increased TLR9 activation, suggesting that PLD3 and PLD4 normally degrade TLR ligands (Figure 2a) [62]. Since TLR9^−/−^ PLD3^−/−^ PLD4^−/−^ mice have more severe disease activity than Unc93B1^−/−^ PLD3^−/−^ PLD4^−/−^ mice, it is likely that TLR7 and potentially TLR13 also contribute to systemic inflammation in double-deficient mice. Intriguingly, PLD4 deficiency on a BALB/c, in contrast to B6, background causes a lupus-like autoimmune disease through a TLR9-dependent mechanism, providing an example of a TLR9-driven model of SLE [63]. Together, these *in vivo* studies suggests that PLD3 and PLD4 normally degrade small stimulatory fragments of DNA and RNA and thereby limit TLR ligand availability.

#### Beyond degradation, nucleases serve as nucleic acid–editing enzymes

Recent research has further delineated the immunomodulatry functions of NA-editing enzymes. NA-editing enzymes are unique nucleases, localized in either the cytoplasm or nucleus, that play critical roles in DNA replication, repair, and transcription. For example, the RNase H2 complex specializes in the degradation of RNA within RNA/DNA hybrids and is therefore responsible for digesting the RNA primers in replication forks, directing ribonucleotide excision repair, and resolving the R-loop structures that form during transcription [64]. Another DNA editing enzyme, SAMHD1, has a dual function as both a deoxynucleotide triphosphohydrolase (dNTPase) and a DNA double-strand break repair enzyme [65]. Dysfunction of RNaseH2 or SAMHD1 leads to genome instability and DNA damage that triggers cGAS-STING pathway and thereby cause AGS [66–68]. Activation of cGAS-STING can further engage additional NA sensing pathways, that then contribute to the pathogenic outcome. For example, in SAMHD1-deficient mice, signals through cGAS-STING pathway prime the RNA sensor MDA5 and amplify its detection of endogenous retroelements-derived dsRNA, resulting in profound IFNb production [69]. In summary, NA-editing nucleases restrict the activity of cytosolic NA sensors to prevent autoinflammation.

#### Nucleases are also required to generate ligands specifically recognized by endosomal Toll-like receptors

The immunoregulatory role of endolysosomal nucleases is complicated by the fact that both DNA and RNA sensing TLRs bind specific nucleic acid degradation products generated by stepwise processing of NA debris. For example, TLR7 contains two ligand binding sites. Site 1 recognizes guanosine and site 2 binds pyrimidine-rich RNA fragments [70]. To generate RNA ligands that can engage both binding sites, RNA in the endosomes are first processed by endonuclease RNaseT2, which preferentially cleave between purines and uridines, generating RNA fragments with 3’ terminal 2’,3’-cGMP. Recent *in vitro* studies have indicated that these RNA fragments that can be further cleaved by exonucleases PLD3 and PLD4 to release the free cGMP, suggesting that the PLD-mediated cleavage can create TLR-binding fragments. As a result, TLR7 responses in RNase T2^−/−^, PLD3^−/−^, or PLD4^−/−^ macrophages are greatly impaired due to lack to proper ligands (Figure 2b) [71,72]. Although RNaseT2-deficient mice develop an autoinflammatory disease, it depends on TLR13 and not TLR7 [73].

TLR9 also contains two ligand binding pockets. Site 1 binds a 5’-xCx motif and site 2 recognizes a CpG motif containing DNA fragments [74]. DNase II can digest long dsDNA into fragments that can engage TLR9 and DNase II–deficient DCs and B cells fail to respond to dsDNA [14,75]. Nevertheless, DNaseII–deficient mice require TLR9-sufficient macrophages for the development of AIH and lupus-like symptoms (see above), and the generation of these TLR9 binding fragments is likely due to a currently unidentified DNase not expressed by B cells or DCs. Whether PLD3 or PLD4 are required to generate TLR9 ligands is unclear since PLD3 and PLD4 appear to limit TLR9 ligand availability, as shown in the PLD3/PLD4 double-deficient mice (see above). The effect of PLD3/4 on TLR9 activation could reflect their ability to degrade existing immunostimulatory fragments [62] or generate immunostimulatory fragments [71].

It is noteworthy that in both DNase II–deficient mice and PLD3^−/−^ PLD4^−/−^ mice, STING contributes to part of the autoinflammatory pathology [55,62,76]. This raises an intriguing question of how accumulation of endosomal DNA (and RNA fragments) gain access to cytosolic sensors. Some potential explanations include rupture of overloaded endosomes and existence of membrane transporters, but the exact mechanisms remain undertermined [77].

## Conclusion — a delicate balance

Our current understanding of autoimmune and autoinflammatory diseases has evolved from a limited number of spontaneously occurring autoimmune-prone mouse strains, to extensive genetic linkage studies, and now the functional characterization of GOF variants of NA sensors and LOF variants of intracellular and extracellular nucleases that promote disease-associated pathologies. Clearly, nucleases maintain a delicate balance in both generating and degrading the NA fragments that engage these receptors. However, much still needs to be learned about how these mutations directly impact specific tissues and cell types to promote chronic sterile inflammation.
